# Information seeking about tool properties in great apes

**DOI:** 10.1038/s41598-017-11400-z

**Published:** 2017-09-07

**Authors:** Manuel Bohn, Matthias Allritz, Josep Call, Christoph J. Völter

**Affiliations:** 1 Max Planck Institute for Evolutionary Anthropology, Deutscher Platz 6, 04103 Leipzig, Germany; 2 School of Psychology & Neuroscience, University of St. Andrews, St. Andrews, Fife UK

## Abstract

Evidence suggests that great apes engage in metacognitive information seeking for food items. To support the claim that a domain-general cognitive process underlies ape metacognition one needs to show that selective information seeking extends to non-food items. In this study, chimpanzees (*Pan troglodytes*) and orangutans (*Pongo abelii*) either had to determine the location of a desired food item or a property of a non-food item (length of a tool). We manipulated whether subjects received prior information about the item’s location or property. During the test, subjects had the opportunity to seek the respective information. Results show that apes engaged in more information seeking when they had no prior knowledge. Importantly, this selective pattern of information seeking applied to food as well as to tools.

## Introduction

Metacognitive processes in nonhuman primates have been studied intensely over the last 15 years. Strategic information seeking by nonhuman primates has been widely regarded as one such process^1–7^, though less inclusive conceptualizations of animal metacognition exist^8, 9^.

Information seeking studies attempt to evaluate the degree to which an animal will search for a desired but hidden object, but only when its location cannot otherwise be determined. Importantly, information seeking studies almost always present subjects with food items as hidden objects. Some have argued that strategic looking for food items, however, may very well reflect natural foraging strategies, devoid of metacognitive processes^10^. In contrast to this account, if strategic information seeking in nonhuman primates extended to desired non-food objects, this would provide evidence that the underlying metacognitive processes are more flexible than previously assumed.

In the present study, we investigated whether information seeking in two species of great apes, chimpanzees (*Pan troglodytes*) and orangutans (*Pongo abelii*), would extend to situations that presented subjects with non-food items, specifically tools. Multiple great ape species have been shown to select tools with regard to functionally relevant properties such as length, weight, thickness, or rigidity^11–15^. In an intriguing recent contribution, Mulcahy^16^ for the first time demonstrated information seeking for tool properties in a study with three orangutans. To acquire food, subjects needed to choose one of two tools (skewers with visible food attached). Importantly, tools were either suitable or broken, and both tools were covered by opaque tubes. In visible trials, tool locations were presented to subjects before choice, whereas in hidden trials they were not. While subjects occasionally looked into the tubes, they did not initially do so significantly more often in hidden trials. Only in a follow-up experiment, in which trials presenting food and trials presenting tools were interspersed, did two of the three subjects exhibit strategic information seeking.

In the present study, great apes had to search for a tool (a raking stick of appropriate length) needed to obtain a reward, or for food directly. Three crucial differences from Mulcahy’s design^16^ characterize the present study. First, unlike in Mulcahy’s study in which pulling the appropriate skewer automatically produced food, the tools in the present study were required to obtain a reward in a different location of the testing room. Strategic information seeking in this context would thus constitute evidence of metacognitive reasoning in a two-step action planning sequence involving actual tool use.

The second major difference was the introduction of two different tool search conditions in which, independent of tool length visibility, the ends of the tools were either visible to subjects at the time of choice, or they were not. We expected subjects to seek information less frequently if at the time of choice tool ends were visible to them, for two different reasons. First, for subjects who approach the site of tool selection in search for a tool, the sheer visibility of protruding tool ends may trigger a predominant pointing response to select any tool before additional information about tool properties is actively sought (inhibition hypothesis). Second, when tool ends are *not* visible to subjects (missing information about tool properties *and* presence), this may result in a stronger sense of perceived uncertainty than when they are visible (missing information about tool properties only), eliciting more information seeking responses in return (uncertainty hypothesis).

Finally, we used a blocked ABA design (tools, food, tools) with a larger sample (N = 14) to investigate in greater depth whether experience with food information seeking is necessary for subjects to exhibit information seeking about tool properties within a given setup.

## Methods

### Subjects

All orangutans at Wolfgang Köhler Primate Research Center who were old enough to participate were included in the study, as was a matched sample of chimpanzees from the same facility. Thus, seven chimpanzees (*Pan troglodytes*, three females, M_age_ = 22.86) and seven orangutans (*Pongo abelii*, five females, M_age_ = 19.43) participated in the study. Across species, subjects were matched in pairs for age and rearing history as much as possible. Subjects participated on a voluntary basis and were never food deprived at any time during the study. All food rewards were given in addition to their regular daily diet. Water was available ad libitum. The study was approved by an internal ethics committee at the Max Planck Institute for Evolutionary Anthropology, Leipzig, Germany. Research was non-invasive and strictly adhered to the legal requirements of Germany. Animal husbandry and research complied with the European Association of Zoos and Aquaria (EAZA) Minimum Standards for the Accommodation and Care of Animals in Zoos and Aquaria and the World Association of Zoos and Aquariums (WAZA) Ethical Guidelines for the Conduct of Research on Animals by Zoos and Aquarium.

### Setup

**Figure 1:**
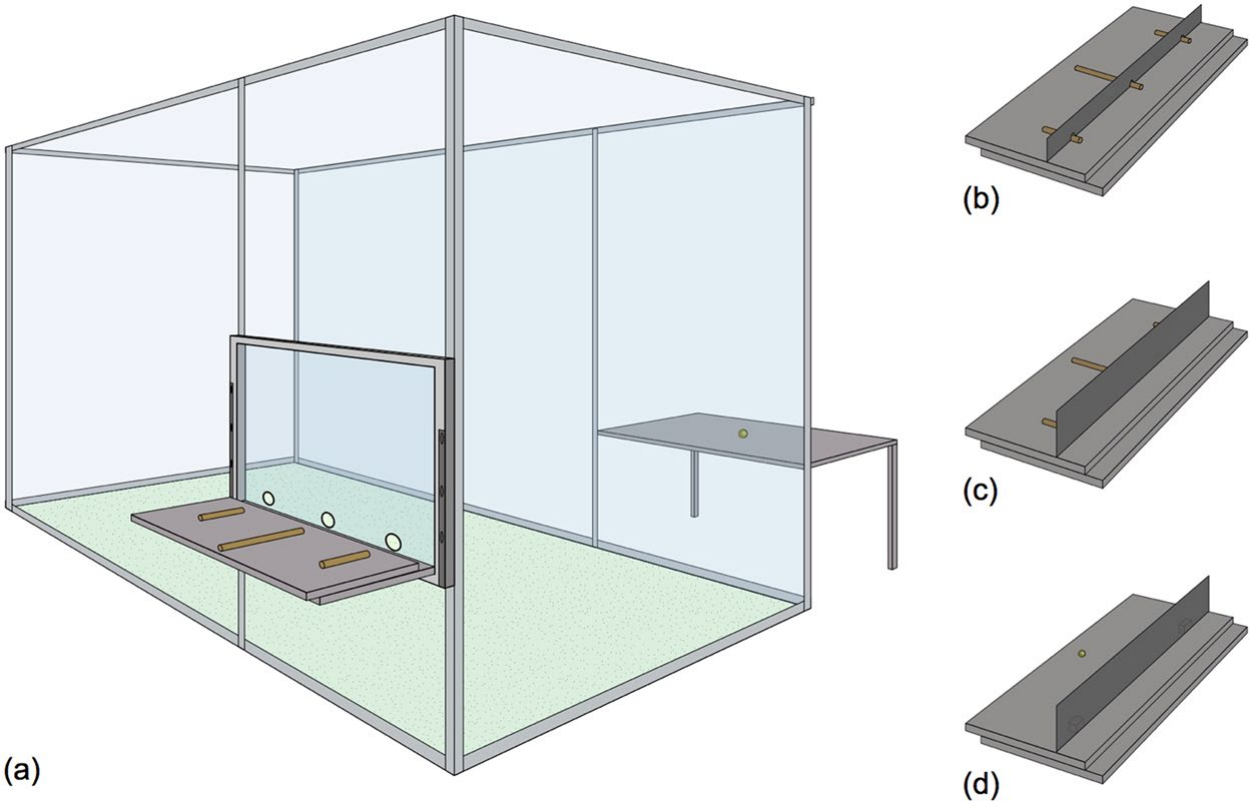
Schematic overview of the general setup (**a**) and the baiting constellations in the tool ends protruding (**b**), tool ends occluded (**c**) and food (**d**) condition. © MPI for Evolutionary Anthropology.

Both species were tested in their familiar sleeping rooms (see Fig. 1). The general setup was modelled after previous studies^17, 18^. Subjects were located in a room where we installed a Plexiglas panel with three evenly spaced holes at the bottom. Behind each hole, on a sliding table (choice table - left side in Fig. 1a), we presented three items from which the subject could choose by pointing through the respective hole. Only one of the items was correct.

In the case of tools, subjects had to choose from three wooden tools: two short (length 10 cm) and one long tool (20 cm). Only the long tool was suitable to obtain an out-of-reach reward placed on a second table (reward table, right side in Fig. 1a). Tools were placed equidistantly from one another (distance 30 cm). The reward (banana pellet) could be accessed by inserting the tool through the mesh at the reward table and bringing it within reach. Rewards were positioned at a distance that required subjects to use the long tool in order to access them (distance approx. 17 cm). The appropriate distance was determined for each individual in a pre-test.

In the case of food, only one item was edible. We placed the food item (a banana pellet) along with two distractor items (small pieces of PVC, same size as the food rewards) on the choice table. These objects had the same distance from one another as the tools. The food item was placed further away from the panel, positioned in the same location as the end of the long tool (Fig. 1d). The table on the right remained empty during food trials.

In some trials, we used small occluders to block the visual access to the tools or the food located on the choice table. Depending on the condition, tools were either occluded completely or only partially. We used a large occluder to block visual access to the choice table between trials.

### Procedure

All subjects received a single training session per condition in which they had to select the correct item (long tool or food item). During training trials, the items were clearly visible to the subject (Fig. 1a). During test trials, the items were placed behind the small occluder (Fig. 1b–d) which required the subject to move close to the panel and slightly upwards in order to gain visual access to the item. The long tool and the food reward could always be seen first when looking over the occluder.

In the beginning of each test session, and between trials, the large occluder covered the choice table. The general trial procedure was the following: Experimenter 1 (E1) lifted the large occluder and called the subject’s attention. After a short delay (depending on trial type, see below), E1 pushed the choice table forward and the subject was allowed to choose by pointing. Subjects could not correct their choices. Subsequently, the experimenter handed over the selected item through the respective hole. In tool test trials (see below), a second experimenter (E2) called the subjects attention to the reward located on the other table before the beginning of the trial. After selecting the long tool, subjects could go over to the reward table and rake in the food. Crucially, we manipulated whether subjects had already seen the items placed on the choice table (visible trials) or not (hidden trials) when making their selection.

#### Visible trials

In visible trials, the items located on the choice table (food or tools) were visible when E1 lifted the large occluder. E1 waited for five seconds (*exposure phase*: starting when the subject attended to the objects on the table) and then put the small occluder in place. After another five seconds (*search phase*), E1 pushed the choice table forward and the subject was allowed to make a choice.

#### Hidden trials

Hidden trials followed the same procedure as visible trials without the exposure phase. That is, after lifting the large occluder, the small occluder was already in place. E1 called the subjects’ attention, waited for five seconds (*search phase*) and pushed the choice table forward.

Note that the physical configuration at the moment of choice was the same in visible and hidden trials. We expected subjects to seek information (move forward and upward, see supplementary video) more in hidden than visible trials.

#### Tool information seeking

In this condition subjects had to select a tool from the choice table in order to access the reward on the reward table. We used two different small occluders. The position from which the tools could be seen through the panel was the same regardless of the occluder. In *tool ends protruding* (TEP) trials we used an occluder (11 × 78 cm) that was placed on top of the tools. The tool ends therefore partly protruded from the occluder and were visible on the subject’s side when the occluder was in place (Fig. 1b). Based on seeing the tool ends alone, it was not possible to identify the long tool. We hypothesized that seeing the tool ends would decrease uncertainty/increase likelihood to prematurely select a tool because the desired object was partially visible. We therefore expected subjects to look less often over the occluder when tool ends were visible. In *tool ends occluded* (TEO) trials, the occluder (17.5 × 78 cm) was placed in front of the tools so that the subject did not see any part of the tool (Fig. 1c). We expected more looks in this condition.

#### Food information seeking

In this condition, subjects had to select the food item placed on the choice table. In test trials, we placed an occluder (the same as in TEO trials) in front of the items. The food item could be seen from the same position, relative to the panel, as the long tool (Fig. 1d).

### Design

We tested subjects in an ABA-design, starting with four sessions (eight trials each) of tool information seeking, followed by two sessions with food, followed by another four sessions with tools. Within the tool condition, each subject received one block with TEP trials (two sessions) and one block with TEO trials (two sessions). The order of TEP and TEO was counterbalanced across subjects. Within each session, we pseudorandomized the order of hidden and visible trials, presenting four of each per session. Trials of the same type never occurred more than twice in a row. In the same way, we pseudorandomized the location of the desired object. Matched pairs across species received the same counterbalancing.

### Coding and analyses

For each trial, we coded the subject’s choice and whether they looked over the occluder during the search phase. The likelihood of selecting the correct tool by chance was one third. We defined *looking* as moving the head into a position from which at least one item behind the occluder was visible. To facilitate coding, a strip of tape on the panel indicated the position above which subjects needed to rise in order to see the objects (see supplementary video). The position of the strip was established by checking the visibility of the objects from the subjects’ point of view. A second coder, blind to the purpose of the study, coded a random selection of 25% test trials. There was high agreement for choice (92.50%; κ = 0.89) as well as for looks (93.93%; κ = 0.76).

We used exact one sample Wilcoxon signed-rank tests to compare subjects’ performance (selecting the correct object) against chance level. This part of the analysis provides a manipulation check: subjects should only perform above chance in visible trials or in hidden trials in which they looked.

We used generalized linear mixed models (GLMM) with a binomial error structure to analyse whether subjects’ looking behaviour was affected by trial type (visible or hidden) and the visibility of the tool ends (TEP or TEO). All models included species and session within condition as control predictors. The random effect structure comprised subject as random intercept and all possible random slopes. All models were fitted in R^19^ using the function *glmer* of the R-package *lme4* ^20^. We used likelihood ratio tests (LRT) to assess whether the inclusion of predictors improved the general fit of a model to the data by comparing models with and without the respective effects^21^. Details about the results and analysis can be found in the supplementary material.

## Results

The dataset is available as part of the supplementary material. Detailed results about subjects’ choice behaviour can be found in the supplementary Table S1. Subjects selected the correct object above chance in visible trials (tool: T^+^ = 105, *p* < 0.001; food: T^+^ = 105, *p* < 0.001) and in hidden trials in which they looked (tool: T^+^ = 55, *p* = 0.002; food: T^+^ = 26, *p* = 0.045) but not in hidden trials in which they did not look (tool: T^+^ = 55, *p* = 0.530; food: T^+^ = 27, *p* = 0.622). This pattern shows that subjects used the information they were exposed to (visible trials), or sought themselves (hidden trials with looks), to select the correct item above chance level.

**Figure 2:**
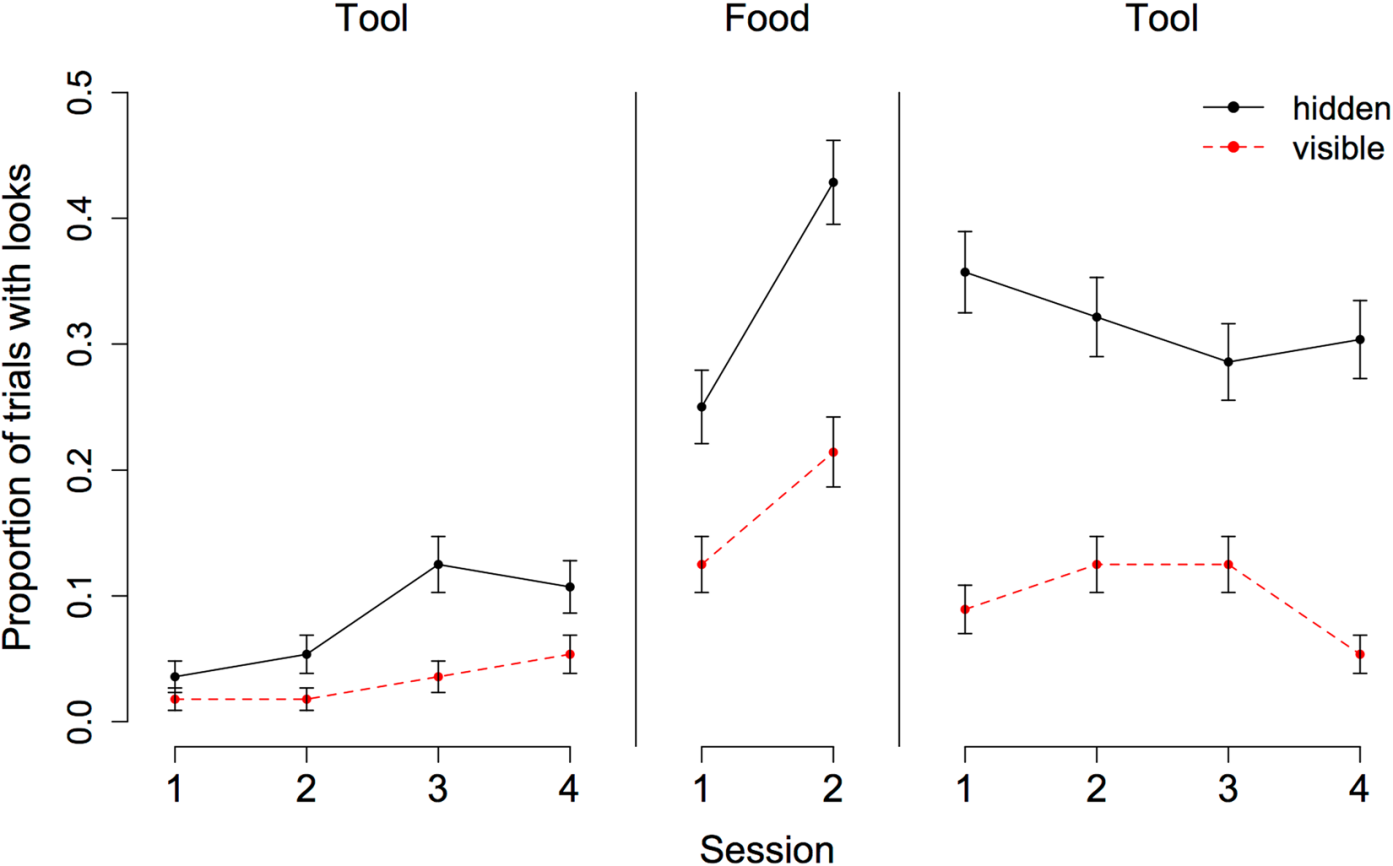
Proportion (+SE) of trials with looks per phase (tool1, food, tool2) and trial type (hidden vs. visible).

Next we analysed subjects’ looking behaviour. Figure 2 shows the proportion of looks per trial type across conditions and sessions. In a first model, we analysed the data for the tool and food condition together. To make food and tool conditions directly comparable, we included only tool trials in which subjects were faced with the same occluder as in the food trials (i.e. TEO trials). In addition to the model structure described above, we included trial type (visible vs. hidden) and condition (tool phase 1, food phase, tool phase 2) as test predictors. Inclusion of these test predictors significantly improved the model fit (χ^2^(3) = 32.82, *p* < 0.001; for the model output, see Table S2). We found an effect of trial type: subjects looked more often in hidden than in visible trials (χ^2^(1) = 15.87, *p* < 0.001). Furthermore, we found an effect of condition (χ^2^(2) = 16.01, *p* < 0.001). Subjects looked more in the food phase compared to the first tool phase (χ^2^(1) = 15.97, *p* < 0.001) but not compared to the second tool phase (χ^2^(1) = 1.38, *p* = 0.238). The control predictors species and session made no significant contribution (species: χ^2^(1) = 0.87, *p* = 0.352; session: χ^2^(1) = 0.54, *p* = 0.463).

In a second model, we specifically addressed the question whether great apes spontaneously look for tool information. Therefore, we analysed all trials of the first tool phase only. This model comprised trial type, species and session as predictors (see Table S3). We found that subjects looked more in hidden trials compared to visible trials (χ^2^(1) = 6.94, *p* = 0.008). We found no effect of species (χ^2^(1) = 0.01, *p* = 0.940) or session (χ^2^(1) = 0.11, *p* = 0.735).

**Figure 3:**
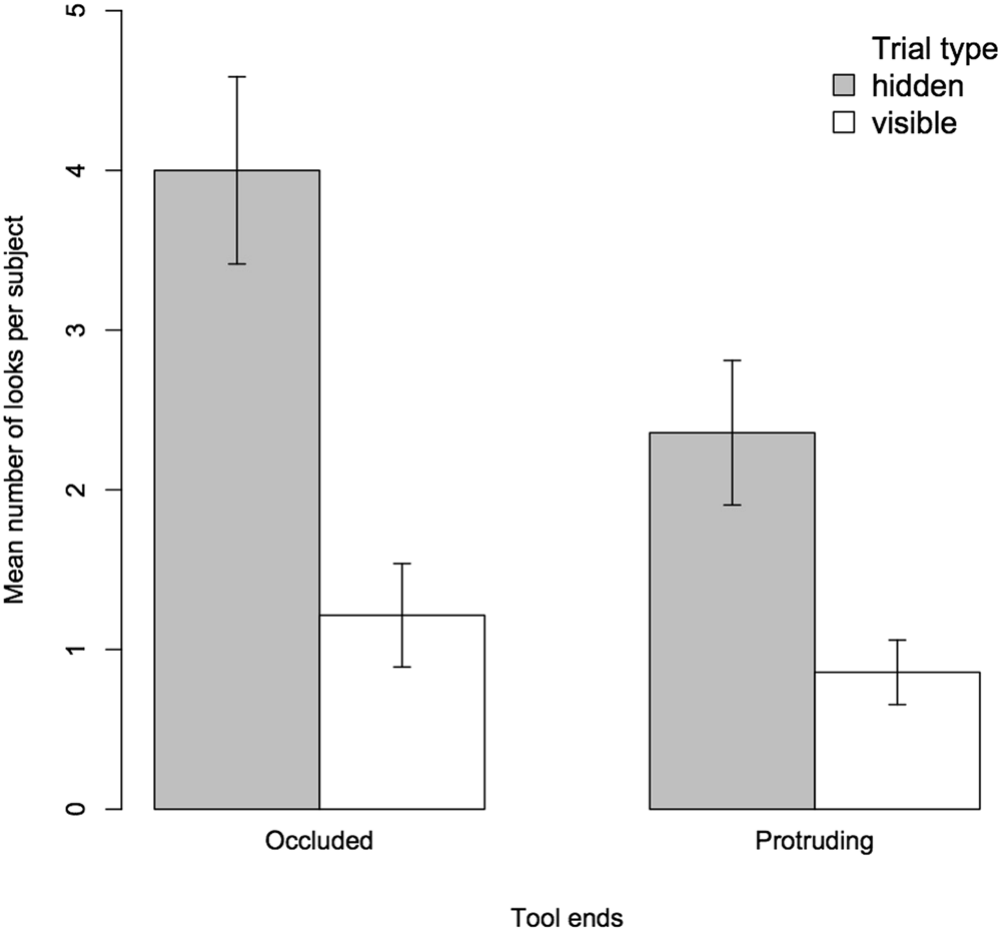
Average number of looks (and SE) per subject in visible and hidden trials for tool ends protruding and tool ends occluded.

In a third model, we investigated the effect of tool end visibility. Therefore, we selected only the data for the tool phases. In addition to trial type, condition (tool phase 1 vs. tool phase 2), species and session, this model comprised tool end visibility as a test predictor. Including this predictor significantly improved the model fit (χ^2^(1) = 7.12, *p* = 0.008; see Table S4). Subjects looked more when tool ends were occluded compared to when they were protruding (see also Fig. 3). Furthermore, subjects looked more often in hidden trials (χ^2^(1) = 13.82, *p* < 0.001) and looked more often in the second tool phase (χ^2^(1) = 5.28, *p* = 0.022). Again, the control predictors species and session made no significant contribution (χ^2^(1) = 0.17, *p* = 0.681; session: χ^2^(1) = 0.12, *p* = 0.730).

## Discussion

Great apes successfully sought information about the location of tools and food items. They predominantly searched for information in trials in which they were missing information (hidden trials) compared to trials in which they had previously been exposed to information (visible trials). Furthermore, apes sought more information when tools were completely hidden compared to when they were only partially hidden. These findings suggest a selective and overall metacognitive search strategy (not exclusively tied to food search).

Previous research has shown that apes search for food when they are uncertain about its location e.g^2, 3, 5^. A recent study further showed that language-trained apes search for information when they are asked to report the identity of the food type^22^. The current study extends these findings to non-food items, thus ruling out that information seeking reflects a natural foraging strategy^10^.

Even though apes did not receive much training with the current tool selection task, one might argue that the long tool acted as a secondary reinforcer. Importantly, such an account is not at odds with our interpretation of the data: nonhuman apes can selectively search for information about objects that have an acquired, task-dependent value to them showing high degrees of flexibility in their search behaviour. One could argue that searching for tools in our study reflects a more generalized foraging strategy that consists of a chain of behaviours that eventually results in obtaining food. While this interpretation appears plausible, it requires an extension of the original natural foraging hypothesis, which was originally formulated to explain searching for food items only^10^, to include information seeking about tools *in the service of* natural foraging (“extended foraging hypothesis”). It is an interesting question for future research whether apes would also engage in information seeking if the sought information is completely independent from foraging (e.g. seeking information about potential threats, mating opportunities etc.).

In previous research^16^, selective information seeking for tools only occurred when tool trials were intermixed with food trials. Searching for tools might have resulted from a carry-over effect from searching for food. In the present study, apes selectively sought information about tools already in the first tool phase, ruling out this alternative explanation. Moreover, because tools and food were separated spatially, our task demonstrates metacognitive reasoning in a two-step action planning sequence. Nevertheless, our results showed that information seeking increased from tool phase 1 to the food phase and remained stable on a higher level in tool phase 2. This suggests that the hiding of food provided a higher incentive for subjects to engage in information seeking than the hiding of tools did. A potential explanation for this pattern could be that in food trials the reward itself was hidden, while it was visible in tool trials. A previous study showed that apes engage in more information seeking when a more valuable reward is hidden^2^. One could argue that food itself has a higher intrinsic value compared to a tool, which is one step away from the food itself, and therefore leads to increased information seeking. However, since we did not include a control group that did not receive the food phase, the increase of information seeking in the food phase is confounded with more experience with the task in general. While the lack of a further increase from the food phase to tool phase 2 speaks against a task experience explanation, future research should address this potential alternative explanation more directly. Interestingly, increased information seeking persisted during tool phase 2: If information seeking in apes was intimately related to food, one would expect that the rate of information seeking would have dropped after the re-introduction of tools.

Finally, as predicted, apes looked more when tool ends were occluded. As noted above, a likely explanation may be that the visibility of tool ends in the TEP condition triggered a predominant pointing response such that subjects simply selected, more often than in the TEO condition, *any* tool before actively seeking additional information about tool properties (inhibition hypothesis). Differing inhibitory control demands may therefore explain lower information seeking rates in this condition. A second, complementary, explanation, could be that apes might have experienced more uncertainty in trials in which they lacked information about tool properties and presence (TEO condition) compared to when they only lacked information about tool properties (TEP condition). Uncertainty about tool presence might be higher in the former case because a longer time interval has passed since the subject last saw tools on the table. In previous studies of food information seeking, longer retention intervals between hiding of the food and test also led to more information seeking^2^. Uncertainty in this sense might have elicited more information seeking for tools in the present study (uncertainty hypothesis). Future studies could attempt to gather additional support for either of these hypotheses by comparing behavioural data that is indicative of different inhibitory control demands (e.g. differing response latencies) or of feelings of uncertainty (wavering, self-directed behaviours)^23^ across conditions in which information is partially vs. fully unavailable.

In summary, great apes spontaneously, selectively and flexibly sought information about tool properties. These results are in agreement with the interpretation that great apes’ metacognitive capacities are domain-general in nature. Future research should further map the boundaries of nonhuman metacognition by examining different contexts (beyond foraging) and types of - missing, incomplete, or potentially surprising - information that can trigger selective information seeking in great apes.

## Electronic supplementary material

Video 1

Supplementary information

Dataset 1
