# Supplementary material for: Information seeking about tool properties in great apes
Source: Sci Rep. 2017 Sep 7;7:10923. doi: 10.1038/s41598-017-11400-z (PMC5589724; doi:10.1038/s41598-017-11400-z)
Supplement: Supplementary file 2 — Supplementary information [file 41598_2017_11400_MOESM2_ESM.pdf]

Information seeking about tool properties in great apes

Manuel Bohn[a], Matthias Allritz[a,b], Josep Call[a,b], Christoph J. Völter[a,b]

[a] Max Planck Institute for Evolutionary Anthropology, Deutscher Platz 6, 04103 Leipzig, Germany

[b] School of Psychology & Neuroscience, University of St. Andrews, St. Andrews, Fife, UK

Supplementary material

Methods

Subjects

Three orangutans previously participated in the study by Mulcahy, Call, and Dunbar (2005).

Setup

Tool ends were located on the sliding table (35 x 78 cm) at a distance of 6 cm from the Plexiglas panel (69 x 48 cm). Subjects could not directly reach them but had to point to make a choice. The holes through which subjects pointed had a diameter of 4 cm. The second table on which the food items had to be raked in was 45 cm high, 75 cm wide and 55 cm deep. In the food condition, the food pellets and the distractors all measured around 3 cm.

Results

In training trials, subjects selected the correct object in 90.47% of tool trials and in 94.64% of food trials. Based on Wilcoxon signed-rank tests this performance was above (33%) chance in both conditions (tool: $T+ = 105$, $p < .001$; food: $T+ = 105$, $p < .001$).

The following tables and figures give detailed information about subjects' performance and looking behavior. As in the main manuscript, p-values for model 1, 2, and 3 as well as S1, S2 and S3 are based on likelihood ratio tests.

Table S1

Average performance (proportion), standard error and test statistic per trial type and looking behavior

Trial type	*N*	M	SE	T^{+1}	*p*[1]
Tool visible	448	0.84	0.04	105	< **.001**
Food visible	112	0.77	0.06	105	< **.001**
Tool hidden, look = 1	89	0.78	0.06	55	**.002**
Tool hidden, look = 0	359	0.36	0.03	55	.530
Food hidden, look = 1	38	0.64	0.13	26	**.045**
Food hidden, look = 0	74	0.31	0.06	27	.622
Tool phase 1 visible	224	0.80	0.03	167	< **.001**
Tool phase 1 hidden, look = 1	18	0.67	0.11	21	**.031**
Tool hidden phase 1, look = 0	206	0.33	0.03	58	.744

[1] based on exact one sample Wilcoxon signed-rank tests; chance level = 1/3

Table S2

Estimates, standard errors and test statistics for Model 1

Predictor	β	SE	χ^2	*df*	*p*
Trial type[1]: visible	-2.03	0.36	15.87	1	< **.001**
Condition[2]: tool1	-2.85	0.64	15.97	1	< **.001**
Condition[2]: tool2	-0.56	0.48	1.38	1	.238
Species[3]: orang	1.83	1.54	0.87	1	.352
Session	0.39	0.23	0.54	1	.463

[1]Reference level: hidden trials
[2]Reference level: food
[3]Reference level: chimp

Table S3

Estimates, standard errors and test statistics for Model 2

Predictor	β	SE	χ^2	*df*	*p*
Trial type[1]: visible	-1.34	0.54	6.94	1	**.008**
Species[2]: orang	0.07	0.99	0.01	1	.940
Session	-0.13	0.39	0.11	1	.735

[1]Reference level: hidden trials
[2]Reference level: chimp

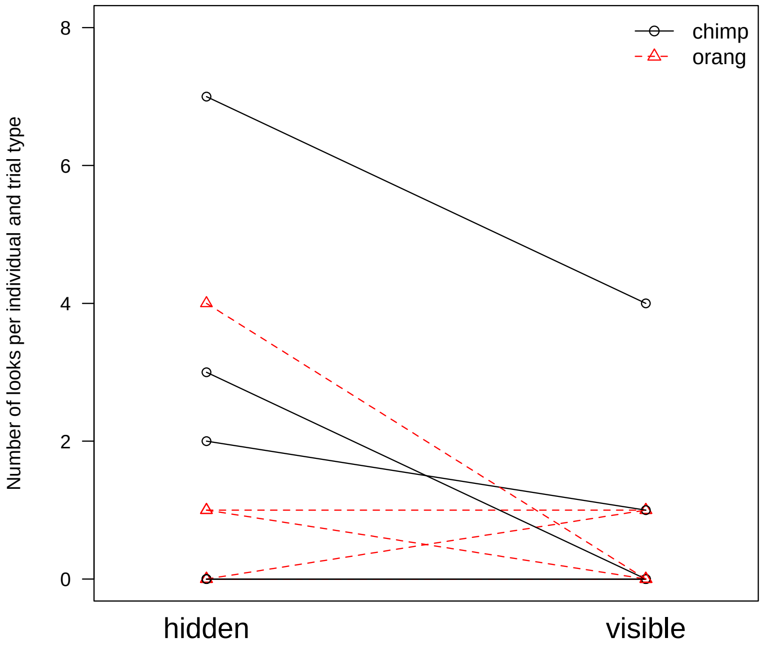

Figure S1. Number of looks per subject and trial type in tool phase1 only. Each subject is represented by a symbol in hidden, a symbol in visible and a line connecting the two.

Table S4

Estimates, standard errors and test statistics for Model 3

Predictor	β	SE	χ^2	*df*	*p*
Trial type[1]: visible	-2.58	0.68	13.82	1	**.008**
Tool ends[2]: protruding	-1.02	0.38	7.12	1	**< .001**
Condition[3]: tool2	1.85	0.61	5.28	1	**.022**
Species[4]: orang	0.47	1.18	0.17	1	681
Session	-0.07	0.21	0.12	1	.730

[1]Reference level: hidden trials
[2]Reference level: tool ends occluded
[3]Reference level: tool1
[4]Reference level: chimp

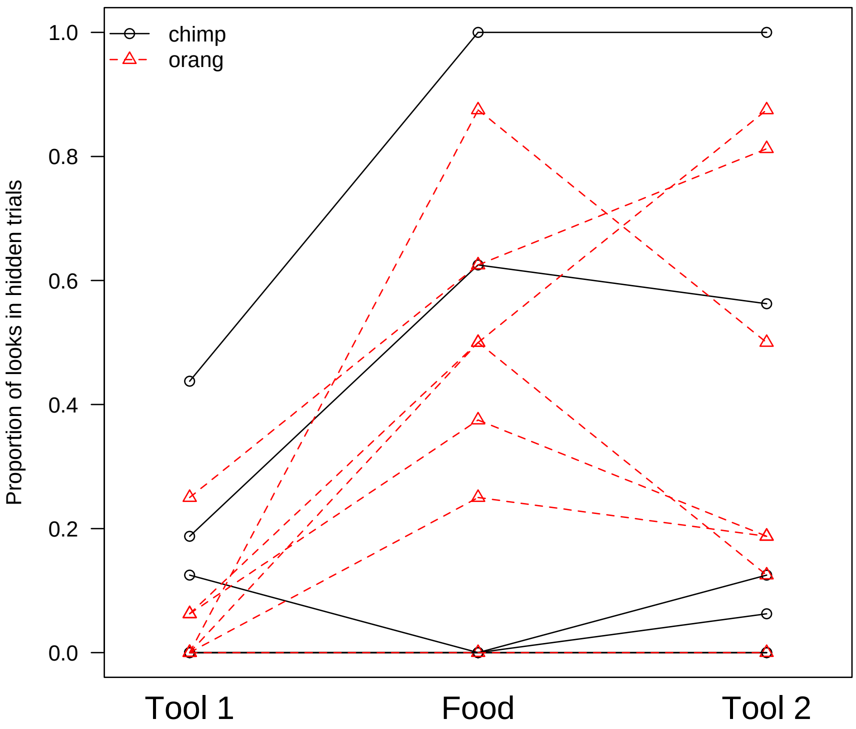

Figure S2. Proportion of looks per subject in hidden trials in each condition. Each subject is represented by a symbol in Tool1, Food and Tool2 and a line connecting the symbols.

Table S5

Estimates, standard errors and test statistics for a version of Model S1 with performance, rather than looks, as dependent variable

Predictor	β	SE	χ^2	*df*	*p*
Trial type[1]: visible	1.95	0.36	17.51	1	< **.001**
Condition[2]: tool1	-0.19	0.27	0.51	1	.473
Condition[2]: tool2	0.32	0.26	1.51	1	.219
Species[3]: orang	-0.35	0.37	0.88	1	.349
Session	0.03	0.13	0.06	1	.800

[1]Reference level: hidden trials
[2]Reference level: food
[3]Reference level: chimp

Table S6

Estimates, standard errors and test statistics for Model S2: Food condition only, look as dependent variable

Predictor	β	SE	χ^2	*df*	*p*
Trial type[1]: visible	-1.79	0.49	10.89	1	**.001**
Species[2]: orang	0.01	2.43	0.00	1	1.00
Session	0.87	0.92	0.53	1	.396

[1]Reference level: hidden trials
[2]Reference level: chimp

Table S7

Estimates, standard errors and test statistics for Model S3: Tool2 condition only, look as dependent variable

Predictor	β	SE	χ^2	*df*	*p*
Trial type[1]: visible	-3.37	0.98	17.00	1	< **.001**
Species[2]: orang	1.60	1.61	1.00	1	.317
Session	-0.19	0.15	1.61	1	.204

[1]Reference level: hidden trials
[2]Reference level: chimp
